# Supplementary figures and images for: Reduced RNA turnover as a driver of cellular senescence
Source: Life Sci Alliance. 2021 Jan 14;4(3):e202000809. doi: 10.26508/lsa.202000809 (PMC7812316; doi:10.26508/lsa.202000809)

## Slide 1
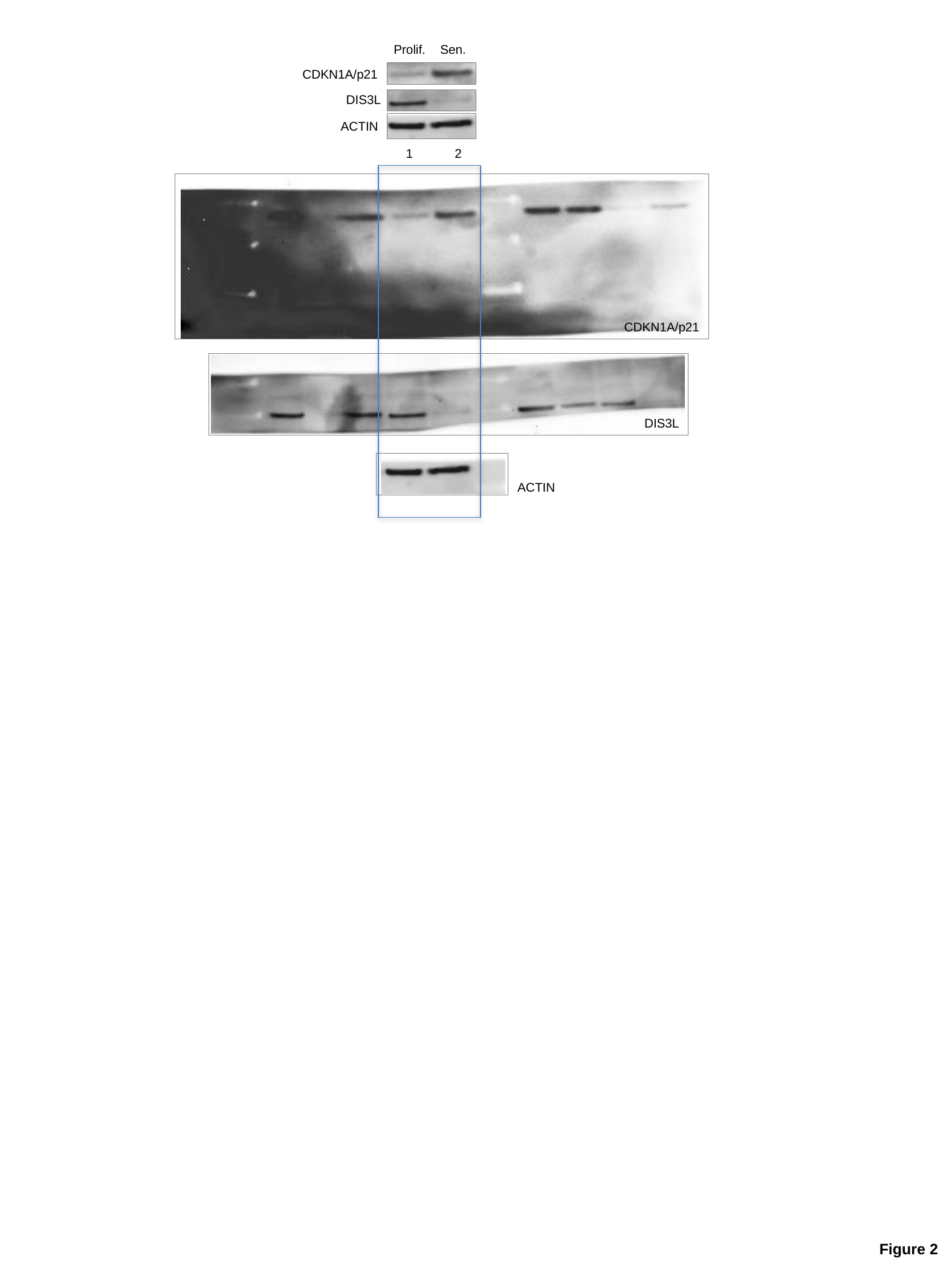

Prolif.
Sen.
CDKN1A/p21
DIS3L
ACTIN
2
1
CDKN1A/p21
DIS3L
ACTIN
Figure 2

Supplement: Supplementary file 1 [file LSA-2020-00809_SdataF2.pptx]
